# Klippel-Trenaunay Syndrome

**Published:** 2013-12-24

**Authors:** Alicia R. Billington, Jehan Shah, Joshua B. Elston, Wyatt G. Payne

**Affiliations:** Plastic Surgery Section, Bay Pines VA Healthcare System, Bay Pines, Fla; and the Division of Plastic Surgery, University of South Florida College of Medicine, Tampa

**Keywords:** Klippel-Trenaunay syndrome, angio-osteohypertrophy, venous malformations, vascular anomalies, macrodactyly

## DESCRIPTION

A mother brings her 8-year-old daughter for evaluation because her daughter's left arm and leg are becoming “more swollen.” History reveals that the child was born with a pale pink mark over her left thigh that has darkened with time. The patient did not walk until 16 months.

## QUESTIONS

1. **What are the classical associations in Klippel-Trenaunay syndrome (KTS)?**
2. **What complications are associated with this disease?**
3. **Should the mother be concerned about her future offspring having a similar presentation?**
4. **What treatment options are available?**

## DISCUSSION

Klippel-Trenaunay syndrome is a rare disease, with estimates of approximately 1 per 30,000 live births.^1^ The disease has a classic triad of capillary malformations, vascular anomalies (classically varicosities such as the persistent embryonic lateral vein of Servelle), and hypertrophy of bony and soft tissues. Patients with Parkes-Weber syndrome have similar presentations as those with KTS and can often be indistinguishable on physical examination. However, advanced imaging has made the differentiation between high- and low-flow arteriovenous malformations (AVMs)—the distinguishing feature—much easier. Low-flow AVMs are seen in KTS and have relatively low morbidity, whereas high-flow AVMs are more appropriately assigned as Parkes-Weber syndrome. Differentiation is important, as high-flow AVMs can cause more serious clinical consequences such as high-output heart failure, more prominent skin manifestations with an increased chance of skin ulcerations, and increased limb-length discrepancies.^2^

The expression and presentation in KTS have a widely varying disease spectrum, from incidental to incapacitating. Symptoms such as pain, skin breakdown, and lymphedema can cause relatively little impairment. However, superficial thrombophlebitis with overlying cellulitis, deep venous thrombosis, and pulmonary embolism can be more severe requiring hospitalization. There are also associated congenital abnormalities, including developmental dysplasia of the hip and syndactyly, roughly 30% of the time.^3^^,^^4^ It is important that the physician is aware that vascular malformations can occur in internal organs and may be the source of major bleeds in anemic patients with KTS.^1^^,^^5^^,^^6^

The etiology of the disease is still under investigation, but it is well accepted that venous abnormalities are not the causative insult. Embryonic mesodermal changes resulting in increased angiogenesis lead to increased vascular flow causing tissue hypertrophy and vascular changes.^7^ Most experts agree that the majority of cases of KTS are due to sporadic polygenic mutations.^6^ A theory of paradominant inheritance seeks to explain KTS by the formation of a lethal mutation in a gene, whereby homozygous embryos do not survive but heterozygous embryos survive and are normal. It is not until a second hit with loss of heterozygosity occurs that phenotypic expression results. The paradominant theory helps explain why rare family links to the disease exist, as well as the mosaicism that can occur in individuals.^5^

A single treatment plan is not recommended, as the specific type and severity of symptoms should guide management in KTS. There are few indications for surgical management. For superficial varicosities, conservative measures (compression therapy) are tried prior to excision or stripping and surgical care is performed only after radiographic confirmation of a patent deep venous system. Regarding limb-length discrepancies, a cutoff of 2.0-cm difference has been suggested for epiphysiodesis.^3^ Sclerotherapy and laser therapy can be used on capillary malformations for cosmetic reasons.^4^ Surgical debulking often fails or worsens symptoms as venous and lymphatic channels are destroyed, leading to further swelling and poor wound healing. Overall, treatment is often not definitive, and 50% of patients reexperience symptoms after surgery despite reported clinical and symptom severity improvement in many patients.^8^

Klippel-Trenaunay syndrome is a rare disease of the vascular and lymphatic system often presenting with a characteristic “nevus flammeus,” or capillary malformation at birth. Vascular malformations and growth abnormalities usually occur in the lower extremities and on the same side of the body. These can lead to hypertrophy of both bony and soft tissues, with hypotrophy being less common.^6^ Most cases are difficult to treat due to high rates of recurrence, but individualized intervention can help manage pain and help prevent serious complications.

## Figures and Tables

**Figure 1:**
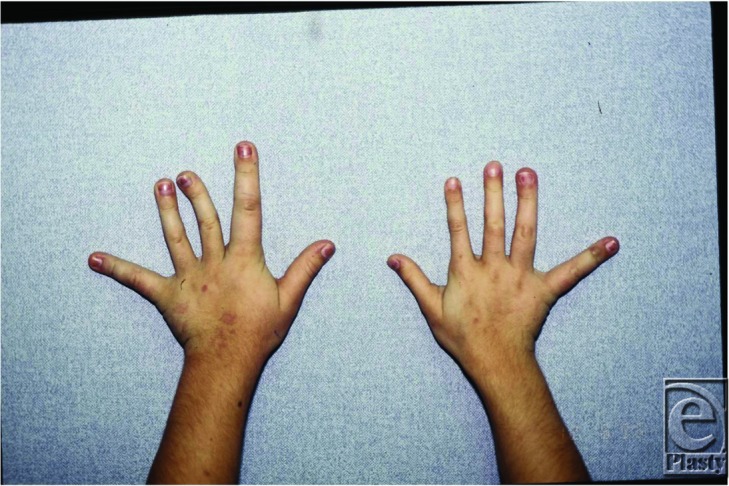
Asymmetric hypertrophy of the left arm and in particular the left first and second digit macrodactyly.

**Figure 2:**
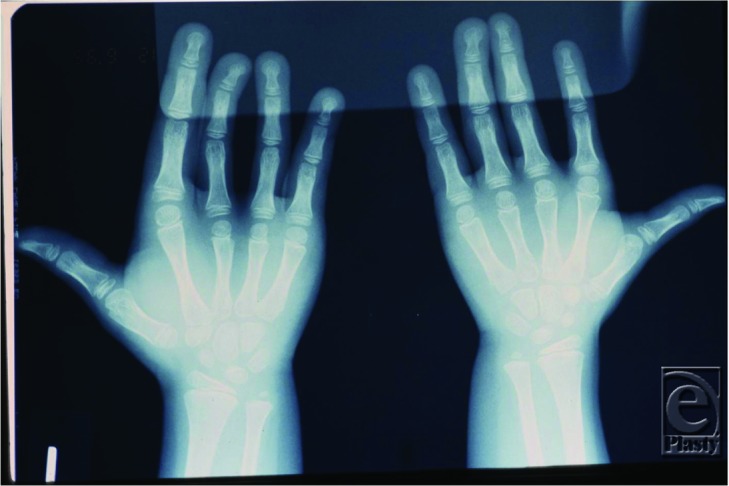
Osteohypertrophy of the metacarpals and phalanges.

**Figure 3:**
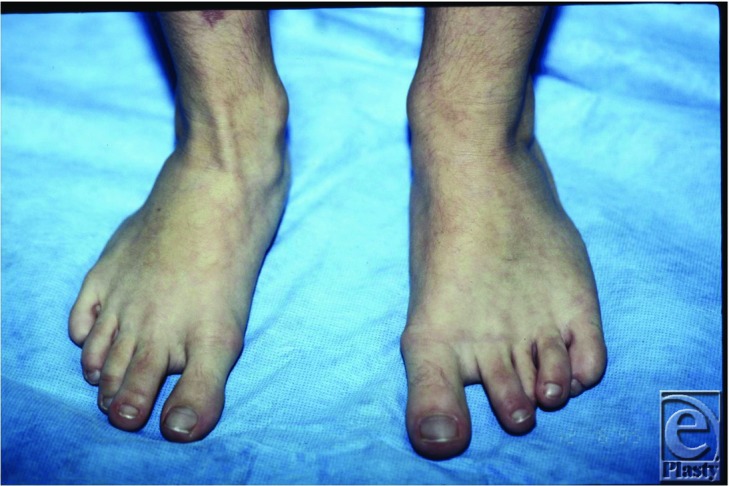
Asymmetric hypertrophy of the left lower extremity.

**Figure 4:**
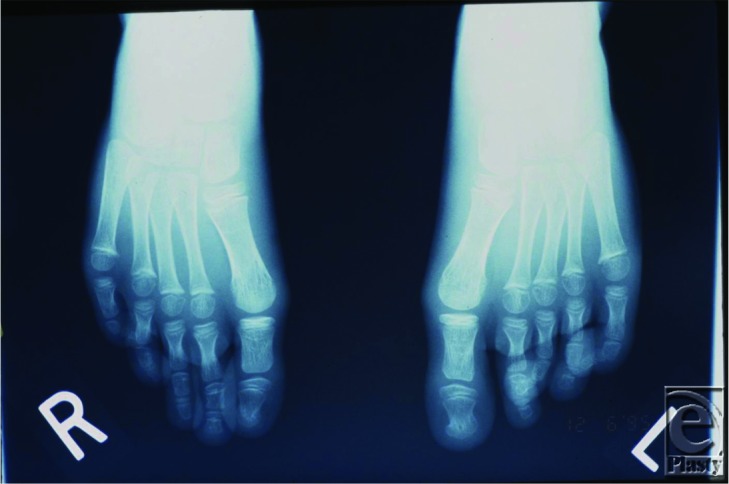
Radiograph of the feet showing osteohypertrophy of the first metatarsal.
